# Melanoma Targeted Therapies beyond *BRAF*-Mutant Melanoma: Potential Druggable Mutations and Novel Treatment Approaches

**DOI:** 10.3390/cancers13225847

**Published:** 2021-11-22

**Authors:** Karam Khaddour, Lucas Maahs, Ana Maria Avila-Rodriguez, Yazan Maamar, Sami Samaan, George Ansstas

**Affiliations:** 1 Division of Medical Oncology, Department of Medicine, Washington University in Saint Louis, Saint Louis, MO 63130, USA; 2 Division of Hematology and Oncology, Department of Medicine, University of Illinois at Chicago, Chicago, IL 60612, USA; lpeter38@uic.edu (L.M.); aavila40@uic.edu (A.M.A.-R.); 3 Division of Hematology and Oncology, Department of Medicine, University of Tishreen Lattakia, Lattakia 2217, Syria; yazan.maamar95@gmail.com; 4 Department of Medicine, American University of Beirut, Beirut 1107, Lebanon; ses19@mail.aub.edu

**Keywords:** melanoma, targeted therapy, precision oncology, *BRAF*, *MEK*, *NF1*, *NRAS*, epigenetic, homologous recombination deficiency, DNA damage repair, tumor suppressor gene, molecular alteration

## Abstract

**Simple Summary:**

The management of unresectable and metastatic cutaneous melanoma has substantially improved with the introduction of molecular targeted therapy (BRAF and MEK inhibitors) and immunotherapy (Immune checkpoint inhibitors). The avenue of precision oncology holds promise in melanomas due to the high rate of somatic mutations that contribute to tumor progression. In this review article, we discuss common mutations and altered pathways that are implicated in melanomagenesis including oncogenic driver mutations, tumor suppressor gene alterations, fusion oncogenes, epigenetic regulators and alterations in the DNA-damage response pathway. We also provide a comprehensive review of promising individualized novel treatment approaches in non-BRAF mutant melanoma.

**Abstract:**

Melanomas exhibit the highest rate of somatic mutations among all different types of cancers (with the exception of BCC and SCC). The accumulation of a multimode of mutations in the driver oncogenes are responsible for the proliferative, invasive, and aggressive nature of melanomas. High-resolution and high-throughput technology has led to the identification of distinct mutational signatures and their downstream alterations in several key pathways that contribute to melanomagenesis. This has enabled the development of individualized treatments by targeting specific molecular alterations that are vital for cancer cell survival, which has resulted in improved outcomes in several cancers, including melanomas. To date, *BRAF* and *MEK* inhibitors remain the only approved targeted therapy with a high level of evidence in *BRAF^V600E/K^* mutant melanomas. The lack of approved precision drugs in melanomas, relative to other cancers, despite harboring one of the highest rates of somatic mutations, advocates for further research to unveil effective therapeutics. In this review, we will discuss potential druggable mutations and the ongoing research of novel individualized treatment approaches targeting non-*BRAF* mutations in melanomas.

## 1. Introduction

**Figure 1:**
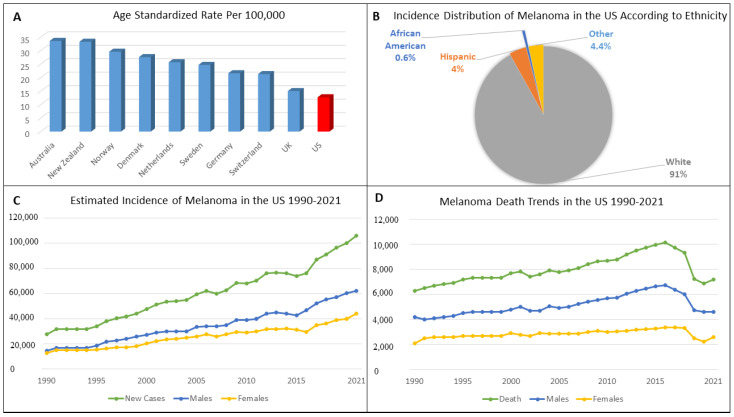
Epidemiology and trends of melanomas. (**A**) Age-standardized rate of melanoma cases per 100,000 in 2018 in selected countries per GLOBOCAN statistics [3]; (**B**) the 2011 incidence distribution among different ethnicities in the US [4]; (**C**) estimated incidence of melanoma cases in the US between 1990–2021 according to the American Cancer Society Statistics [6]; (**D**) estimated melanoma annual deaths in the US between 1990–2021 according to the American Cancer Society Statistics [6]. GLOBOCAN: Global Cancer Observatory.

Melanoma remains the deadliest skin cancer despite substantial advances achieved in its management. According to the International Agency for Research on Cancer, the estimated global new cases of melanoma in 2020 was 324,635, and the number of new deaths was 57,043 [1]. The age-standardized death rate due to melanoma is one death per 100,000 persons globally [2]. Incidence rates vary according to the geographical region and the Fitzpatrick skin phenotype, with the highest incidence rates reported in Australia, New Zealand, Northern Europe, and North America (Figure 1A) [3]. The incidence in the African-American population is one case per 100,000, compared to 22.1 cases per 100,000 in white patients (Figure 1B) [4]. The projected increase in the incidence of melanomas in the United States is estimated to reach 56.1 in males and 36.2 in females by 2036, which represents a three- to four-fold increase in melanoma incidence [5]. This trend of an increase in incidence and mortality in melanoma patients in the last three decades in the United States represents a major challenge in healthcare due to the burden of the disease (Figure 1C,D) [6].

Locally advanced and metastatic melanomas constitute 13% of newly diagnosed cases (SEER database statistics 2021). Despite the small proportion of the metastatic version of the disease, the mortality rate remains high and lags behind the number of newly diagnosed cases. The treatment of metastatic melanomas has substantially improved in the last decade, owing to the collaborative work that unveiled the role of oncogenic driver mutations and the immune system dysfunction involved in melanoma progression. The identification of the *BRAF^V600E/K^* as an oncogenic driver of somatic mutation in melanomas resulted in the development of the targeted treatment of the *BRAF* gain-of-function mutation (GOF) [7]. Similarly, the discovery of essential checkpoint receptors (including *CTLA-4* and *PD-1/PD-L1*) and their role in immune evasion in cancers, including melanoma, reinvigorated the effort to develop immunotherapies that have proved crucial in prolonging survival [8,9,10,11,12].

The first *BRAF* inhibitor in melanomas was vemurafenib, which demonstrated efficacy in *BRAF^V600E/K^* mutant melanomas in the BRIM-3 phase 3 randomized clinical trial that demonstrated the overall survival (OS) advantage compared to dacarbazine, with an objective response rate (ORR) of 48% [13]. Following the BRIM trial, several *BRAF* inhibitors emerged as effective treatment options in *BRAF^V600E/K^* mutant melanomas. *BRAF* inhibitors were combined with *MEK* inhibitors in clinical trials due to their synergism, which can delay tumor progression and increase acquired resistance [14]. Three clinical trials (COMBI-d, coBRIM, and COLUMBUS) demonstrated superior outcomes in terms of the ORR and progression-free survival (PFS) for the combinations dabrafenib + trametinib, vemurafenib + cobmitinib, and encorafenib + binmetinib, compared to vemurafenib monotherapy in unresectable and metastatic melanomas harboring *BRAF^V600E^* or *BRAF^V600K^* mutations [15,16,17].

Unlike targeted therapy in *BRAF^V600E/K^* mutant melanomas, immune checkpoint inhibitors (ICI) demonstrated improved clinical outcomes in melanoma patients regardless of the presence of an oncogenic mutational signature or biomarker. Several phase 3 randomized clinical trials have demonstrated a high ORR and prolonged PFS and OS in front-line and recurrent metastatic melanomas, regardless of their *BRAF* mutation status [18,19,20,21].

The trials of the *BRAF/MEK* inhibitors were limited to patients with *BRAF^V600E^* and *BRAF^V600K^* mutations and who showed an ORR between 60 and70%, which indicated that 30–40% of *BRAF* mutant melanoma patients did not respond to the targeted therapy. Similarly, the ICIs showed an ORR in melanomas of 35–40% with single checkpoint inhibitors, and 58% with combined nivolumab and ipilimumab [18,19,20,21]. Accordingly, 40–60% of melanoma patients did not respond to ICI. In addition, the PFS rate at 5 years was 15–20% with *BRAF/MEK* inhibitors, and 21–36% with ICIs [22,23,24,25]. Taken together, these results demonstrate the unmet need for further effective therapeutics in melanoma patients, due to the substantial rate of non-responders to either targeted therapy or ICI, as well as the high rate of progression after the initial response.

The distinctive genetic alterations in melanomas provide an advantage for cancer cells to operate in an aggressive and unpredicted manner. For example, the frequency of somatic mutations is the highest in melanomas among solid and hematological malignancies (>100 somatic mutations per megabase) with the exception of non-melanoma skin cancers [26]. The characterization of the mutational landscape in melanomas from The Cancer Genome Atlas (TCGA) revealed specific molecular and genetic alterations including GOF mutations, loss-of-function (LOF) mutations (nonsense, splice, frameshift) in tumor suppressor genes, recurrent hotspot mutations, and copy number variations (amplifications and deletions) as well as different epigenetic, transcriptomic, and proteomic changes [27]. This has led to a new genomic classification of melanomas, which identifies subsets with distinct genetic signatures [28].

The diverse mutational landscape in melanomas represents an insufficiently explored territory of multiple potential actionable genetic alterations. Of importance, despite the high rate of mutations in melanomas, many of these are considered passenger mutations (i.e., they are not vital for melanoma progression). Research efforts are ongoing to elucidate the pathogenic implications of the vital molecular alterations to develop novel treatments. In this review, we will discuss common and infrequent/rare mutations in melanomas. We will also discuss current novel therapeutic approaches that are underway to target non-*BRAF* mutant melanomas. This review is tailored to cutaneous melanomas and, where relevant, we will mention acral and mucosal melanomas. Emphasis is drawn on the importance of including patients with refractory melanomas in clinical trials whenever possible.

## 2. The Mutational Landscape in Metastatic Melanomas

**Figure 2:**
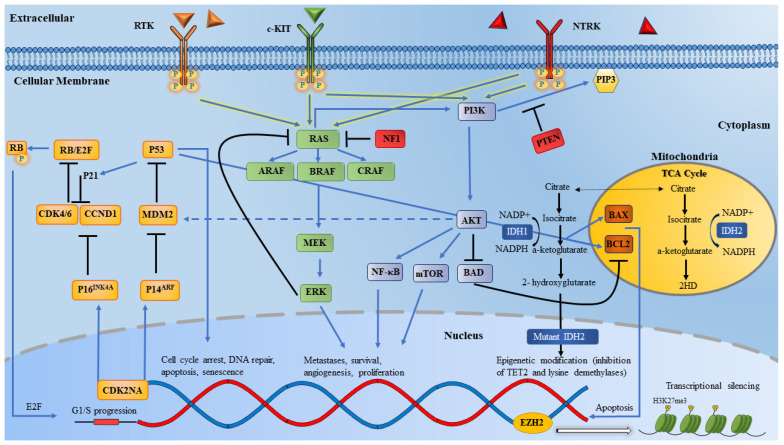
Major signaling pathways in melanomas. The most essential pathways in melanomagenesis are the MAPK pathway (in green) and the PI3K pathway (violet). The MAPK pathway is activated by receptor tyrosine kinase and G-protein-coupled receptors. This activates *RAS* proteins, which in turn activates *MEK*, and then *ERK. ERK* can then translocate to the nucleus and phosphorylate transcriptional factor substrates involved in cell survival. *NF1* negatively regulates *RAS* proteins which inhibits downstream *RAS* signaling. The PI3K pathway can be activated by *RAS* or through the inactivation of *PTEN*. PI3K can activate several pathways (*BAD, NF-kB*) and *AKT* pathways, which in turn leads to phosphorylation of *mTOR,* which leads to increased cellular proliferation. The *CDKN2A* pathway is another essential network in melanomas (yellow). *CDKN2A* encodes two inhibitory variants of the G1–S phase. These inhibitors (tumor suppressors) are *P16^INK4A^*, which can bind to *CDK4/6*, preventing them from interacting with *CCND1* and *RB* phosphorylation. When phosphorylated, *RB* can release an *E2F* factor which leads to G1–S cell cycle progression. *P14^ARF^* can bind to *MDM2* which is responsible for p53 degradation. Epigenetic regulators can be altered in melanomas. *IDH1/2* are enzymes that convert isocitrate to a-ketoglutarate. *IDH1/2* mutations can lead to the formation of the oncometabolite *D2H* that can alter and silence several key regulator genes (although the exact role of *IDH* mutations in melanomas has not been elucidated). Hotspot mutations and amplifications in *EZH2* can lead to aberrant methylation of *H3K27*, leading to dysregulation of transcriptional factors. Alterations in *c-KIT* and *NTRK* fusions (although rare in melanomas) can contribute to melanomagenesis through downstream signaling of several pathways, including MAPK and PI3K, to drive cellular proliferation. Abbreviation: RTK: receptor tyrosine-kinase; NTRK: neurotrophic tyrosine receptor kinase; PIP3: phosphatidylinositol 3; PI3K: phosphoinositide 3-kinase; PTEN: phosphatase and tensin homolog; NF1: neurofibromatosis type 1; RAS: rat sarcoma; RB: retinoblastoma; E2F: E2 factor; CDK4/6: cyclin-dependent kinase 4/6; CCND1: cyclin D1; MDM2: mouse double minute 2 homolog; MEK: mitogen-activated protein kinase; CDK2NA: cyclin-dependent kinase inhibitor 2A; ERK: extracellular signal-regulated kinase; AKT: ak strain transforming; NADP+/NADPH: nicotinamide adenine dinucleotide phosphate; IDH1: isocitrate dehydrogenase 1; NF-kB: nuclear factor kappa-light-chain-enhancer of activated B cells; mTOR: mammalian target of rapamycin; BAD: BCL2 associated agonist of cell death; BAX: BCL2-associated X protein; BCL2: B-cell lymphoma 2; IDH2: isocitrate dehydrogenase 2; EZH2: enhancer of zeste homolog 2.

**Figure 3:**
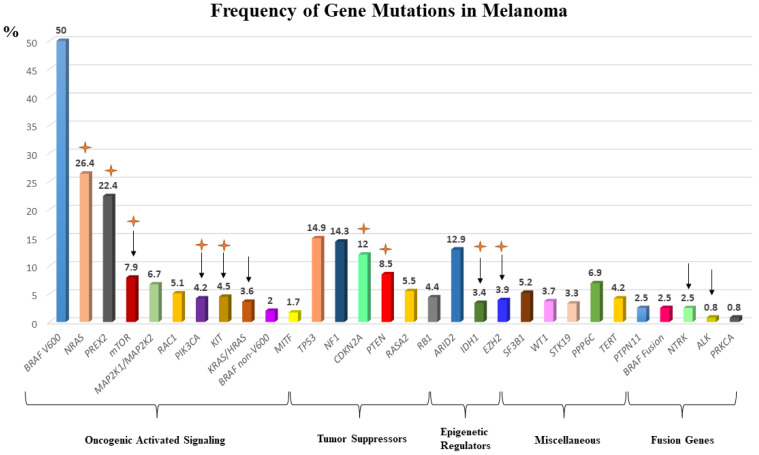
Frequency of common somatic mutations in melanomas are estimated based on Vanni et al.’s analysis of the frequency of somatic mutations from different studies [33]. Fusion gene frequencies were obtained from non-TCGA database, and the frequency varies among different studies. Arrows indicate the presence of FDA-approved inhibitors in cancers harboring mutations for the specific gene in non-melanoma cancers. Orange asterisks indicate the presence of ongoing trials in melanoma patients with mutation-specific alterations or melanoma patients receiving targeted therapy for the specific mutant gene. TCGA: the cancer genome atlas; FDA: food and drug administration.

The two most commonly altered cellular pathways in melanomas are: (1) the mitogen-activated protein kinase (MAPK) pathway, and (2) the phosphatidylinositol 3-kinase (PI3K) pathway. Together, the MAPK and PI3K pathways form important signaling cascade networks that transmit extracellular signals intracellularly to regulate cell division and differentiation. Putative oncogenic activating mutations in *BRAF* (with a frequency of 40–60% in melanomas), *NRAS,* and *KIT,* as well as gene fusions, can signal through the MAPK and PI3K pathways, leading to uncontrolled cellular growth, proliferation, and survival (Figure 2). These mutations appear to be mutually exclusive with rare exceptions [29]. Further recurrent mutations have been identified through major collaborative work using high-resolution whole genome/exome sequencing by the Pan-Cancer Analysis of Whole Genomes Consortium, TCGA, and the cBioPortal, among other platforms (Figure 3) [28,30,31,32]. Recurrent mutations can be GOF mutations such as: *MAP2K1/MAP2K2*, *mTOR*, *KRAS/HRAS*, *RAC1,* and *TERT*, while LOF mutations occur in tumor suppressor genes such as: *NF1*, *PTEN*, *CDKN2A,* and *TP53*. Likewise, copy number variations (CNV) have been identified in certain genes in association with melanomas, such as in *PTEN*, *CDKN2A,* and *KIT*. In addition, alterations in epigenetic regulators like *EZH2*, *ARID2,* and *IDH1/2,* as well as genomic instability caused by mutations in the DNA damage response (DDR) genes, can lead to the altered transcription of key gene regulators in melanomas. In addition, mutations in melanomas can be either driver or passenger mutations. Driver mutations are able to drive malignant transformations in melanoma cells by virtue of the constitutive activation of growth signaling pathways. Conversely, passenger mutations can occur by chance, and do not confer the survival advantage to tumor cells. The distinction between these two types of mutations is challenging, but is important for the development of effective targeted therapy.

Tumorigenesis in melanomas is not driven by one single genetic alteration, but rather it requires collaboration between several altered pathways. However, *BRAF*, *NRAS,* and *NF1* mutations were found to be prevalent and highly oncogenic [34,35]. As a result, melanomas have been classified into four distinct genetic subsets based on the molecular signature (*BRAF* mutant, *NRAS* mutant, *NF1* mutant, and triple negative) [28]. The improved sequencing techniques helped identify novel mutations and alterations (overexpression/amplification) that can drive melanomagenesis, such as mutations in the microphthalmia-associated transcription factor (*MITF*), Rac family small GTPase1 (*RAC1*), serine/threonine kinase-19 (*STK19*), and *RAB7* [27,28]. It is noteworthy that the implementation of *BRAF* and *MEK* inhibitors in clinical practice is a testimony of the impressive improvement in outcomes by targeting a subset of oncogenic driver mutations in melanomas. Nevertheless, the lack of targeted therapy in non-*BRAF* mutant melanomas, the eventual progression during treatment with *BRAF/MEK* inhibitors, and the high rate of somatic mutations in melanomas relative to other malignancies advocates for further research into their biological effects on tumor progression [33,36].

## 3. Targeting Activating Mutations in the *KIT* Oncogene

*KIT* was first described in 1987 as a receptor tyrosine kinase (RTK) encoded by the proto-oncogene *c-KIT* localized on chromosome 4 [37,38]. The receptor has the characteristic structure of other RTKs, which includes an N-terminal signal peptide, an extracellular segment, a transmembrane domain, and a C-terminal intracellular segment. *KIT* is an RTK type III, which is characterized by five immunoglobulin-like repeats in the extracellular domain. The C-terminal intracellular segment contains the kinase domain, which is subdivided into an N-terminal segment that corresponds to an ATP-binding site, and a C-terminal segment that corresponds to the kinase autophosphorylation site [39,40].

*KIT* activation occurs by the binding of the stem cell factor ligand (SCF, also known as the *KIT* ligand), a growth factor that stimulates the survival, differentiation, and proliferation of several cell types, including melanocytes [41,42]. The ligand binds to the three immunoglobulin-like domains in the N-terminal portion of the extracellular segment and causes the dimerization of the receptor by enabling a homotypic interaction between the two domains closer to the cellular membrane. This causes the phosphorylation of the tyrosine domains intracellularly, as well as subsequent transmembrane signal transductions [40,43,44]. Once activated, *KIT* promotes the survival and development of hematopoietic stem cells, germ cells, and melanocytes. The most important signaling pathways in *c-KIT-*derived melanomas are the PI3K/*AKT* and the MAPK pathways [45,46,47].

Mutations in *c-KIT* are relatively infrequent in melanomas and were first reported by Went et al. who sequenced 36 tumors that were strongly positive for *KIT* by immunohistochemistry (IHC). These included two melanomas, of which one had a transition mutation of leucine to proline in codon 576 (L576P) of exon 11. It was later found that L576P is the most common mutation in *c-KIT* in melanomas and that most mutations occur in exons 11 and 13 [47,48,49]. In addition, mutations of *c-KIT* seem to be more common than copy amplifications in melanomas and most of them occur in the kinase domain or in the juxta-membrane domain. These alterations seem to vary in frequency depending on the melanoma subtype and are mutually exclusive to *BRAF* and *NRAS* mutations, with rare exceptions. Both the mutations and amplifications of *c-KIT* are uncommon in cutaneous melanomas (3–4%) but are enriched in acral (8.6–36%) and mucosal melanomas (9.6–39%) (*KIT* mutations are enriched in sinonasal mucosal melanomas, as opposed to anogenital) [50,51,52,53].

The standard of care for metastatic melanomas with *c-KIT* mutations is immunotherapy [54]. There are no prospective studies evaluating the response of *KIT* mutant melanomas to immunotherapy, with one retrospective study showing an ORR of 20% in patients who received anti-*CTLA-4* agents and had *KIT* mutations in exons 2, 11, 13, and 17. The same study reported an ORR of 35% with anti-PD1 immunotherapy in patients with *KIT* mutations in exons 2, 10, 11, 13, and 17 [55]. The authors described that the patients had similar response rates to ICI, regardless of the exon affected [55].

Treatment approaches with tyrosine kinase inhibitors (TKIs) targeting *KIT* mutations have been conducted in several clinical trials in relapsed and refractory melanomas. Imatinib, which has multiple therapeutic targets including *BCR-ABL, KIT,* and the platelet-derived growth factor receptor (*PDGFR*), was approved for the treatment of gastrointestinal stromal tumors (GIST) (the majority of these tumors have the *KIT* mutation) [56]. The first trials evaluating imatinib in melanomas were negative and noted significant treatment toxicity. However, these trials did not select patients with *KIT* mutations for enrollment [57,58]. Following that, one phase 2 trial enrolled 21 patients with metastatic melanomas expressing at least one protein tyrosine-kinase (*c-KIT, PDGFR, c-abl*, or *abl-*related genes) to receive imatinib. One patient with the highest *c-KIT* expression had a dramatic response, four patients (three expressing *c-KIT*) had a stable disease, and the remaining patients had disease progression [59]. This trial has paved the way for further investigation into the role of TKIs in *KIT* mutant melanomas.

The first trial investigating the targeted therapy of *KIT* mutations was reported in 2011 and included a total of 51 melanoma patients who received imatinib. This trial demonstrated two complete responses (CR) lasting 53 and 89 weeks, and two partial responses (PR) lasting 12 and 18 weeks, among 25 evaluable patients. The reported ORR was 16% with a median progression-free survival (PFS) of 12 weeks and median overall survival (OS) of 46.3 weeks. Patients who achieved a CR or a PR had *KIT* mutations in exons 11 and 13 in acral or mucosal melanomas [60]. Two other trials and one retrospective study evaluated imatinib in this setting and the results were similar, as shown in Table 1. However, no patients achieved a CR as reported in the first trial. In trial 2, 10 patients out of 43 had a PR, of which nine had a *KIT* mutation in exon 11 or 13, and one patient had a *KIT* amplification. In trial 3, seven patients out of 25 achieved a PR, six of which had exon 11 or 13 mutations and one who had an exon 17 mutation [61,62]. The largest study evaluating imatinib was a retrospective study with 78 patients that did not report a CR, but there were 17 PRs (11 in patients with exon 11 or 13 mutations, two with multiple mutations, one with an amplification, and one in exons 9, 17, and 18) [63].

**Figure 4:**
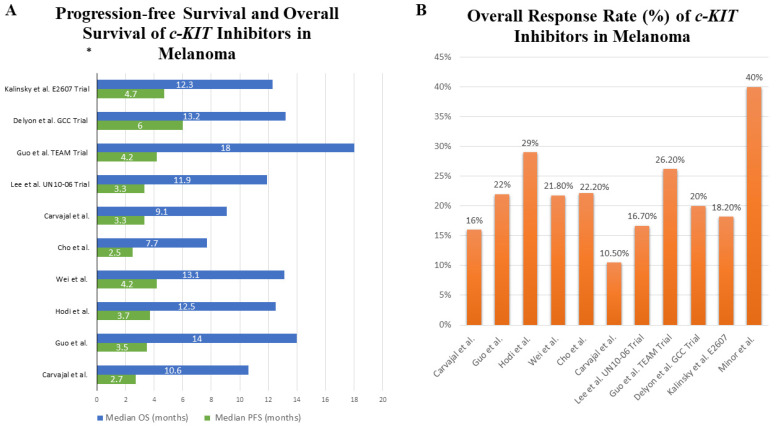
Clinical trials and retrospective study results of *c-KIT* inhibitors in melanomas [60,61,62,63,64,65,66,67,68,69,70]. (**A**) Median progression-free survival and overall survival in melanoma patient cohorts treated with different *c-KIT* inhibitors. These trials and retrospective studies must not be compared as patient inclusion criteria, trial designs, and *KIT* mutation subtypes are different. (**B**) Overall response rates in melanoma cohorts treated with *c-KIT* inhibitors. * Kalinsky et al. study analyzed PFS and OS in melanoma patients with *KIT* mutations involving exons 11 and 13 only [69]. PFS: progression-free survival; OS: overall survival.

Trials with a similar design were conducted using newer TKIs, including nilotinib, sunitinib, and dasatinib, and are summarized in Table 1. These trials demonstrated similar results in terms of response rates, PFS, and OS (Figure 4A,B). Most responses in the studies were noted in patients with *KIT* mutations in exons 11 and 13. Other *KIT* alterations, such as mutations in other exons, amplifications, and overexpressions, may have lower response rates [60,61,62,63,64,65,66,67,68,69,70]. One phase 2 trial using dasatinib did not include exclusively patients with *KIT* mutations. The ORR was 5% (a total of two PRs, one in a patient with a *c-KIT* exon 13 mutation, and one in a patient with a wild-type *c-KIT*), which emphasizes the concept of using these TKIs in *c-KIT* mutant melanomas only [71].

Some case reports suggested the efficacy of other TKIs in melanomas with *KIT* alterations. For example, a patient with anal mucosal melanomas and a *KIT* mutation (Val560Asp) in exon 11 had a complete response to sorafenib with temozolomide that lasted 5 months [72]. Another case report demonstrated PR (including an intracranial disease) that lasted for 3 months in a patient with a primary esophageal mucosal melanoma and a *KIT* mutation in exon 11 treated with masitinib [73].

In general, the responses reported in all previous studies were short-lived, suggesting that other pathways may contribute to resistance. One possible mechanism of resistance explored is the activation of the *MET* receptor. One study reported the case of a patient with a metastatic melanoma of unknown origin that harbored a *KIT* mutation, N822Y, in exon 17. The patient received dasatinib for *KIT* inhibition and crizotinib for *MET* inhibition and had a response sustained for 34 months. The authors demonstrated in vitro that the addition of the hepatocyte growth factor (*MET* ligand) to the melanoma cell lines with the *KIT* mutation was able to increase cell viability despite the presence of dasatinib, which was reversed by the addition of crizotinib [74].

There is also preclinical evidence that ponatinib may have a role in the treatment of *KIT* mutant melanomas. One study obtained patient-derived tumor xenograft cells from *KIT* mutant melanomas and tested the efficacy of *KIT* inhibitors in vitro and in vivo. Ponatinib had a stronger affinity for *KIT* and was a more potent inhibitor when compared to imatinib, suggesting the need for future studies with newer TKIs in *KIT* mutant melanomas [75].

Some studies have also looked into combining a TKI with other systemic therapies. One phase 1/2 trial tested the combination of 800 mg of imatinib daily and 10 mg/kg of bevacizumab every 2 weeks in 23 patients with metastatic melanomas. One patient (4%) had PR and there was a stable disease in seven patients (30%). The median PFS was 7.7 weeks, and five patients remained on the study for more than 4 months [76]. Another study combined imatinib and ipilimumab in 35 patients with different advanced malignancies and demonstrated one PR in a patient with melanoma and a *KIT^L576P^* mutation in exon 11. The duration of the response observed was 10 months [77]. The combination of pembrolizumab and imatinib was reported in the case of a patient with a metastatic melanoma and double *KIT* mutations (V559 and N822I). The patient had oligometastatic disease in the lung and achieved a CR after 6 months of therapy, which lasted for 12 months [78].

Several clinical trials are testing novel TKIs and combinations of TKIs with other systemic therapies for melanomas, such as NCT02071940, which involves evaluating PLX3397 (plexidartinib, a multi-target TKI) in advanced *KIT* mutant acral and mucosal melanomas. Another trial (NCT01738139) is investigating the combination of ipilimumab and imatinib in advanced solid tumors including metastatic melanomas. NCT04598009 will evaluate binimetinib and imatinib in patients with stage III and IV *KIT* mutant melanomas. In addition, NCT02571036 is a phase 1 trial that will evaluate DCC-2618 (ripretinib, a TKI designed to inhibit specifically *KIT* and *PDGFR-A* kinases) in patients with advanced cancers, and the trial NCT04771520 is currently recruiting patients with advanced or metastatic solid tumors with *c-KIT* or *PDGFR-A* mutations to receive avapritinib (BLU-285).

In summary, *KIT* is evolving as an important therapeutic target in advanced melanomas. Currently, the use of TKI is reserved for the second or later line treatment in *KIT* mutant melanomas. The role of *KIT* inhibition in patients with advanced melanomas and *KIT* alterations is rapidly expanding. So far, the response rates have been low and most of the responders have sensitized mutations in exons 11 and 13. Clinical trials are currently evaluating more potent inhibitors and the combination of current inhibitors with immunotherapy. More studies are needed to further evaluate the characteristics of patients that achieve a response, the mechanism of resistance development, and the role of combining immunotherapy and *MET* inhibitors with *KIT* inhibitors.

## 4. Targeting Activating Mutations in *RAS* Oncogenes

### 4.1. NRAS

#### 4.1.1. *NRAS* Biology

The *NRAS* oncogene was first identified in a melanoma cell line in 1984 [79]. Since then, several efforts have been conducted to develop targeted therapy strategies for *NRAS* mutant melanomas. *NRAS* is part of the *RAS* proteins (*NRAS, KRAS* and *HRAS*) and is a small plasma membrane-associated guanosine 5′-triphosphate (GTP)-binding protein. The most frequent oncogenic mutation (>80%) is a point mutation at position 61, leading to the substitution of leucine with glutamine. This point mutation results in impaired GTPase activity and the locking of the *RAS* protein into its activated (GTP-associated) conformation. The activated *RAS* subsequently transmits the signal from *RTK* to several downstream transduction pathways involved in growth, motility, cell-to-cell signaling, differentiation, and survival.

In normal melanocytes, MAPK signaling occurs selectively through *BRAF* rather than *CRAF*, as the cyclic AMP pathway activation promotes protein kinase A (*PKA*) mediated inhibition of *CRAF*. In *NRAS* mutant melanomas, the inhibition of *PKA* signaling (that prevents *CRAF* inactivation) leads to the negative feedback inhibition of *BRAF* and promotes *CRAF*-mediated MAPK signaling instead [80].

Upstream effectors controlling *NRAS*, such as *RTK*, have been identified. More recently, Yin et al. identified a critical kinase (*STK19*) upstream that phosphorylates *NRAS* on the S89, promoting oncogenic *NRAS* signaling. This led to the development of a selective *STK19* inhibitor with preclinical results in vitro and in vivo exhibiting inhibitory effects on *NRAS* mutant melanomas [81].

#### 4.1.2. The Frequency in Melanomas and Characteristics

*NRAS* mutations account for 26% of all mutations present in melanomas registered in TCGA [28]. Comparing the genetic alterations in 126 melanomas, Curtin et al. identified that 81% of melanomas on skin without chronic sun damage had mutations in *BRAF* or *NRAS* [82]. *NRAS* mutations occur at a higher rate in the older population, where the median age is 55.7 years compared to 49.8 years in patients with the *BRAF* mutation [83]. In addition, *NRAS* mutations are present predominantly in upper extremities and in one third of nodular melanomas [84,85].

In a retrospective study of 677 patients, 82.4% of *NRAS* mutations accounted for substitutions in positions 60–61 and, most frequently, a glutamine to arginine/lysine/leucine/histidine substitution at position 61 (*Q61R/K/L/H*) [83,86]. Uncommonly, 20% of all *NRAS* mutations are due to a glycine to aspartic acid/arginine substitution in positions *12–13 G12* (*G12D*), and *G13* (*G13R, G13D*). Burd et al. demonstrated in vitro and in “knock-in” mouse models that the expression of the *NRAS* mutation at codon 61 drives melanoma formation with increased melanomagenecity compared to the *NRAS* mutation at codon 12. Therefore, this explains the predominance of *NRAS* mutations in *Q61R* [87].

#### 4.1.3. The Prognostic Impact of *NRAS* in Melanomas

The prognostic impact of *NRAS* mutations in melanomas is controversial. A large retrospective review of patients with melanomas reported that *BRAF* and *NRAS* mutations were more likely to have CNS involvement at the time of diagnosis, and *NRAS* mutant melanomas were an independent predictor of shorter survival times in metastatic melanomas compared to *BRAF* mutant and wild-types [83]. In addition, *NRAS* mutation is associated with the deepest Breslow and Clark levels of invasion and patients tend to present with regional metastases compared with wild-type tumors. In the setting of stage III disease, Ellerhorst et al. reported no difference in survival between mutant (*BRAF* and *NRAS*) and wild-type melanomas; however, in this study the mutation pattern was only obtained from the primary site and metastases were not sequenced [85].

A European retrospective multicenter analysis (*n* = 364) comparing ICI therapy between *NRAS* mutant and wild-type melanomas showed a shorter median OS in patients with *NRAS* mutations (21 months compared to 33 months) despite similar response rates. In this study, loco-regional or distant metastases were used for mutation patterns [88].

In contrast, a large retrospective analysis (*n* = 656) of a Clinico-Genomic Database evaluating outcomes in a real-world setting demonstrated that after the first line of immunotherapy, the median OS of *NRAS* mutant melanomas was 44.9 months compared to 38.6 months in *BRAF* mutants, 27.1 months in *NF1* mutants, and 19.8 months in triple wild-type melanomas [89].

Similar findings were reported by Mangana et al. in a smaller study (*n* = 101) evaluating anti-*CTLA-4* in *NRAS* and *BRAF* mutant melanomas, however, results did not reach statistical significance [90].

#### 4.1.4. Targeting Upstream Effectors of *NRAS*

Targeting *RTK* remains a challenge in melanomas. Early studies have been conducted in combination with downstream inhibitors with mixed results. A phase 1 study with an antibody targeting *ERBB3* in combination with trametinib in *NRAS* mutant or wild-type melanomas was terminated in 2020 (NCT03580382). The *MET* receptor tyrosine kinase, activated in *NRAS* mutant cancers, has been targeted with the oral inhibitor tivantinib. A phase 1 study in combination with sorafenib was extended to a cohort of patients with melanoma. Among the eight patients with *NRAS* mutations, there was one CR, one PR, and two stable diseases (SD) [91].

#### 4.1.5. Targeting *NRAS*

The direct targeting of the ligand binding sites on all *RAS* proteins remains challenging due to their high affinity for GDP, GTP, and suspected high toxicity. Given *RAS* is essential in cell signaling, the deletion of all three *RAS* proteins results in embryonic lethality in mouse models and no cellular proliferation in vitro. Other strategies have been studied, such as small binding compounds inhibiting the *SOS1*-mediated nucleotide exchange on *RAS* and the reduced phosphorylation of the downstream kinases *ERK* and *AKT*. However, the compounds are not mutant-selective inhibitors and no specific strategy targeting *NRAS* has been developed [86].

#### 4.1.6. Post-Translational Targets in *RAS* Proteins

*RAS* proteins undergo a lipid post-translational modification in order to get access to the effectors in the membrane compartments. Farnesylation is one of the key post-translational modifications which is catalyzed by the enzyme Farnesyltransferase (*FT*). Therefore, *FT* inhibitors have been developed as post-translation targets to reduce *RAS*- mediated downstream activation. In melanomas, the *FT* inhibitor lonafarnib, in combination with sorafenib, showed preclinical activity against tumor cell growth in vitro and in vivo. In a phase 2 clinical trial, R115777 (an oral selective *FT* inhibitor) did not show evidence of clinical activity in a cohort of 14 patients with melanoma [92,93].

#### 4.1.7. Targeting the Downstream Effectors of *NRAS*

The activation of the *RAS/RAF/MEK/ERK* signaling pathway through *NRAS* mutations occurs in 15–20% of melanoma cases. In vertebrates, there are three *RAF* proteins called *ARAF, BRAF*, and *CRAF,* and they are the initial effectors in the kinase cascade. Dorard et al. demonstrated that the concomitant ablation of *BRAF* and *CRAF* in *NRAS* Q61K mutant mouse melanoma models resulted in a blockage of tumor growth, providing evidence that the MAPK pathway is an important downstream effector of oncogenicity in *NRAS* mutant melanomas [94].

The downstream oncogenic effect of *NRAS* mutations is mainly driven by *CRAF,* which subsequently signals to *MEK,* and is associated with cAMP signaling dysregulation [95]. Therefore, the selective inhibition of *BRAF* in *NRAS* mutant melanomas is not an adequate approach, as it can induce the paradoxical activation of *RAF* proteins and the concomitant ablation of *BRAF* and *CRAF*, leading to the emergence of resistant cells showing the *ARAF*-dependent reactivation of *ERK* [94].

Solit et al. emphasized the concept that *RAF/MAPK* signaling is dispensable for the oncogenic activity of *NRAS* mutant melanomas, and they suggested that a single-agent therapeutic strategy may be insufficient in *RAS* mutant tumors [96].

Pan-*RAF* inhibitors, such as belvarafenib, which can target *BRAF^V600E^,* the *BRAF* wild-type, and *CRAF* have shown anti-tumor efficacy in *NRAS* mutant melanomas in a phase 1 dose escalation study with an ORR of 44% [97]. Subsequently, in a phase 1b study in combination with cobimetinib (a *MEK* inhibitor), it showed an ORR of 38.5% with median PFS of 7.3 months. Another pan-*RAF* inhibitor (PRi, Amgen Compd), in combination with trametinib, demonstrated anti-proliferation properties in vitro in *NRAS* mutant melanomas [98].

Targeting *MEK* is currently the most developed strategy in *NRAS* mutant melanomas. First-generation *MEK* inhibitors (selumetinib) failed to show positive outcomes in patients with unselected *BRAF/NRAS* mutations, as well as patients with *NRAS* mutations [99]. Similarly, a phase 2 trial double-blind study that analyzed the *NRAS* mutation status retrospectively did not show an improvement between selumetinib with docetaxel vs. a placebo [100].

Second- and third-generation *MEK* inhibitors are the most advanced in this setting, especially binimetinib, which was evaluated in the NEMO study. This study was a randomized phase 3 multicenter trial comparing binimetinib vs. dacarbazine in *NRAS* mutant melanoma patients that showed an improvement in their median PFS at 2.8 months in binimetinib groups, vs. 1.5 months in the dacarbazine group. However, no difference was observed in OS [101]. Pimasertib, a second-generation *MEK1/2* inhibitor, also showed an improvement in the PFS over dacarbazine (13.0 vs. 6.9 weeks) in a phase 2 study in *NRAS* mutant melanomas; however, the OS was similar [102].

Given that *NRAS* activates both the MAPK and PI3K pathways, the combination of the *MEK* inhibitor (trametinib) with the PI3K/*mTOR* inhibitor has shown to enhance cell growth inhibition in vitro [103]. This combination was found to be synergistic, and was effective in inducing tumor reduction in a nude mouse *NRAS* mutant xenograft tumor model [104]. Nevertheless, a non-randomized multicenter phase 2 study using trametinib in combination with GSK2141795 (a pan *AKT* inhibitor) did not yield significant clinical activity in *NRAS* mutant melanomas [105].

The *CDKN2A/CDK4/6* pathway has a role in the G1–S transition in the cell cycle, which is dysregulated in *BRAF* and *NRAS* mutant melanomas. In addition, *NRAS* activation causes increased cyclinD1 (*CCND1*) expression and the upregulation of *CDK4/6*. Alterations in *CDKN2A* and in *CCND1* are present in *NRAS* mutant melanomas in 70% and 10% of cases, respectively [28,106]. A combination of ribociclib (a *CDK4/6* inhibitor) and a *MEK* inhibitor (binimetinib) was evaluated in a phase 1b/2b trial in 63 patients with *NRAS* mutant melanomas, obtaining an ORR of 19.5% (*n* = 41) and a PFS of 3.7 months in the phase 2 expansion [107].

Finally, *MEK* inhibitors have also been studied in combination with ROCK inhibitors (GSK269962A) in vitro and in vivo, showing the suppression of the growth of *NRAS* mutant melanomas. In this case, ROCK 1 and 2 are Rho GTPase-activated serine/threonine kinases implicated in tumor cell proliferation [108]. To date, there have been no clinical trials with this combination.

Despite the lack of clinically effective targeted therapy in *NRAS* mutant melanomas, studies are ongoing to address novel treatment approaches and are summarized in Table 2.

### 4.2. Targeting KRAS/HRAS

Mutations in *KRAS* and *HRAS* occur at a low frequency in melanomas, occurring at 1.7% and 1.9%, respectively [33]. Targeting *KRAS* and *HRAS* mutations in melanomas is not currently being pursued due to the lack of insight on the biological impact in melanomagenesis. Nevertheless, the projected number of new melanoma cases with *KRAS*/*HRAS* mutations is substantial and is around 2600 persons per year [109]. Therefore, further research is essential to investigate the role of these alterations in melanomas. Mutations in *KRAS* have been regarded as undruggable until a recent novel molecule (sotorosib), which targets the *KRAS^G12C^* mutation, demonstrated efficacy in phase 1 and 2 trials in non-small cell lung cancer [110]. Of interest, in the phase 1 trial which included multiple solid tumors with *KRAS^G12C^* mutations, there was one melanoma patient with *KRAS^G12C^* [111]. The patient received 960 mg of sotorasib daily and achieved PR within 2 months of starting treatment. The duration of response was 5.6 months which was followed by disease progression.

## 5. Targeting Tumor Suppressor Gene Alterations

Tumor suppressor genes are responsible for maintaining normal cellular division. Tumor suppressor genes can halt cellular growth, induce apoptosis, and provide a checkpoint state to allow for the repair of damaged DNA, or can induce apoptosis if genomic integrity is not achieved. The most commonly mutated tumor suppressor genes identified in melanomas are: *NF1*, *TP53*, *CKDN2A,* and *PTEN* [28]. The targeted treatment of tumor suppressor gene alterations in cancer has proved challenging despite extensive research efforts [112]. Nonetheless, several promising approaches are being investigated in cancers with tumor suppressor gene alterations.

### 5.1. Targeting NF1 in Melanomas

The neurofibromatosis-1 protein *NF1*, which is encoded by its parent tumor suppressor gene (*NF1)* on chromosome 17, operates by the negative regulation of the MAPK and PI3K pathways. This function is mediated by the GTPase-activating protein-related domain which converts active *RAS*-guanosine triphosphate (RAS-GTP) to the inactive guanosine diphosphate form, leading to the downregulation of the *RAS* cascade, which prevents uncontrolled cellular growth [113]. The majority of *NF1* mutations are LOF nonsense mutations which deprive the cell of the negative regulation of growth pathways. The frequency of *NF1* mutations in melanomas is approximately 15%, with a higher frequency observed in older patients, chronic sun damage lesions, desmoplastic melanomas, and *BRAF*/*NRAS* wild-type melanomas (a frequency of 70%) [114,115]. In addition, *NF1* mutations can co-occur with *BRAF*/*NRAS* mutations and can mediate the resistance to *BRAF* inhibitors. To date, no studies have identified an effective targeted therapy in *NF1* mutant melanomas. However, preclinical evidence suggests that *NF1* mutant melanomas are dependent on *MEK* signaling and may be sensitive to *MEK* inhibitors [116,117]. Recently, Py et al. reported the efficacy of trametinib (a *MEK* inhibitor) in a patient with an immunotherapy-refractory melanoma with *BRAF*/*NRAS* wild-types and mutations in *NF1* and *PTPN11* [118]. The patient had a PR, including the regression of brain metastases, with a duration of response lasting 5 months prior to disease progression. In a phase 1 study using trametinib in advanced melanomas, trametinib did not demonstrate efficacy in the majority of patients with *BRAF*/*NRAS* wild-types, but one patient with an *NF1* mutation had a stable disease [119]. No specific clinical trials are currently ongoing in patients with *NF1* mutant melanomas, but several trials are evaluating targeted therapy in *NF1* mutant solid tumors, including melanomas: (1) The MATCH screening trial evaluating trametinib for the treatment of *NF1* mutant refractory solid cancers (NCT02465060); (2) the MatchMel trial which includes a cohort with *NF1* mutant refractory tumors receiving trametinib, sorafenib, or everolimus (NCT02645149); and (3) the use of RMC4630 (a potent *PTPN11* inhibitor) and cobmitinib in solid tumors with *NF1* mutations (NCT03634982).

Finally, an approach involving the stabilization of the *NF1* protein by reducing its degradation has been suggested to enhance sensitivity to trametinib in melanoma cell lines. In this study, Alon et al. demonstrated that *NF1* could be rescued through the inhibition of calpain1 (*CAPN1*), which is a calcium-dependent neutral cysteine protease that plays a role in *NF1* degradation, therefore providing a blockage of *RAS* activation in melanoma cells [120]. However, prospective clinical trials are still needed to study the safety and efficacy of this approach. To this end, the current standard of care in melanoma patients with *NF1* mutations remains the use of ICI.

### 5.2. Targeting TP53

*TP53* is an essential tumor suppressor gene which maintains the integrity of the cellular genome. Inactivating mutations of *TP53* are the most prevalent mutations in solid tumors [121]. In melanomas, mutations in *TP53* occur at a frequency of 15% with the majority being LOF due to the inactivating missense mutations, as well as a simultaneous loss of heterozygosity (LOH) deletion in chromosome 17 [28,122]. *TP53* mutations can co-exist with other driver mutations, such as with *BRAF* and *NRAS* [123]. The loss of the regulatory function of p53 (the transcribed protein of *TP53*) is a late event in melanomas and is caused by several mechanisms, including structural mutations in *TP53*; the overexpression of the mouse double minute 2 (MDM2) oncoprotein, which is responsible for the ubiquitination and degradation of p53; mutations in *P14^ARF^*, which lead to an overexpression of MDM2; and the overexpression of the MDM2 homolog murine double minute x (MDMX) which inhibits p53 [124,125]. The research efforts to overcome the LOF of p53 is based on two different approaches: (1) the restoration of mutant p53 function, and (2) the restoration of wild-type p53 function through the inhibition of negative regulators such as MDM2 and MDMX. Several preclinical studies have demonstrated anti-tumor effects in wild-type *TP53* tumors using novel inhibitors of negative regulators, such as inhibitors of MDM2/MDMX, in melanoma cell lines and animal xenografts. However, there are no current trials in humans evaluating the safety and efficacy of this approach [126,127,128,129]. A phase 1 study of *AMG-232* (an MDM2 inhibitor) and trametinib evaluated the safety and maximum tolerated dose in patients with metastatic cutaneous melanomas with wild-type *TP53* [130]. Of the 15 evaluable patients, 13% (2/15) patients had a PR, 73% (11/15) had a SD, and 13% (2/15) had a progressive disease.

The ability to target p53 mutant cancers by restoring p53 function was recently supported by two studies in hematological malignancies using eprenetapopt (APR-246), which is a small molecule prodrug administered intravenously. When activated, eprenetapopt can bind to cysteine residues in mutant p53, leading to thermodynamic stabilization as well as shifting the equilibrium of p53 into its active conformation. However, its efficacy was only reported in myeloid dysplastic syndrome (MDS) and acute myeloid leukemia (AML) when used in combination with azacitidine [131,132].

### 5.3. Targeting CDKN2A and CDK4/6 Networks

The cyclin-dependent kinase inhibitor 2A (*CDKN2A*) plays an important role in cell cycle regulation through its complex networks, which converges with other important genes such as *TP53* and *RB*. Germline mutations in *CDKN2A* are an established risk factor for the development of hereditary melanoma syndromes, and increase the risk of hereditary melanomas by at least 10-fold [133]. The *CDKN2A* is located on chromosome 9p21 and encodes two distinct proteins, *p16^INK4A^* and *p14^ARF^*. The main function of *p16^INK4A^* is to regulate the G1–S phase and cellular senescence by binding to *CDK4/6,* leading to the interruption of the interaction with *CCND1* and the phosphorylation of *RB*. This, in turn, locks the *RB/E2F* in its inactive form, preventing the release of the transcription factor *E2F* and G1–S phase progression. *P14^ARF^* exerts its tumor suppressor effects through binding to MDM2, which leads to the inhibition of p53 degradation [134].

Alterations in the *CDKN2A* pathway in melanomas can occur due to either copy number changes (deletions of *CDKN2A*, the amplification of *CCND1*, and the amplification of *CDK4/6*), mutations in *CDKN2A* or *CDK4/6,* and promoter hypermethylation of *CDKN2A* [27]. The frequency of mutations of *CDKN2A* is approximately 12% and deletions can be present in up to 28% of cutaneous melanomas [33].

Several trials are ongoing to assess the safety and efficacy of drugs targeting the dysregulation of the *CDKN2A/CDK4/6* pathways using *CDK4/6* inhibitors such as palbociclib, ribociclib, and abemaciclib. Preclinical evidence suggests the antitumor synergistic efficacy of *CDK4/*6 inhibitors, with *BRAF/MEK* inhibitors and *MEK* inhibitors, in *BRAF* and *NRAS* mutant melanomas, respectively [135,136,137]. The anti-tumor efficacy of single agent *CDK4/6* inhibitors is unknown in melanomas bearing specific dysregulations in the *CDKN2A* pathway. However, some evidence exists regarding their potential anti-tumor effects. For example, in a phase 1 dose escalation trial of abemaciclib in solid tumors including melanoma, there was one PR and six patients with stable diseases among 26 patients who had melanoma [138]. Of interest, the patient who achieved a partial response was found to have a copy number loss of the *INK4* locus. Unfortunately, a phase 2 study of abemaciclib in 23 melanoma patients with intracranial metastases showed 0% objective responses, but patients were selected irrespective of the presence of the *CDKN2A* pathway alteration [139]. Ribociclib was studied in a phase 1 trial in patients with advanced solid tumors. Among 132 patients, there were three patients with melanomas, with one achieving a PR and one achieving a stable disease [140]. Of interest, the patient with the partial response had *CCND1* amplification. In regards to Palbociclib, a phase 1 trial in China evaluated its efficacy in patients with acral lentiginous melanomas with *CDKN2A* pathway alterations [141]. Among the 15 patients enrolled in this trial, there were three documented responses (an ORR of 20%) with one achieving a PR with palbociclib monotherapy. Given the lack of insight into the efficacy of the *CDK4/6* inhibitor monotherapy in melanomas, and the possible synergy and efficacy when combined with other inhibitors in *BRAF* and *NRAS* mutant melanomas, most of the current ongoing trials are evaluating combination *CDK4/6* inhibitors with other targeted therapies [142].

## 6. Targeting Fusion Gene Alterations

Fusion genes refer to chromosome rearrangements during cell mitosis by joining separate segments of different genes [143]. The rearrangement process can happen due to chromosome translocation, inversion, deletion, or duplication. The newly fused chimeric proteins can lead to the constitutive activation of the kinase domains of several pathways, including MAPK, PI3K, and phospholipase C (*PLC*), that promote cell survival in both hematological and solid malignancies [144]. The most common characterized gene fusions in cancer include *ALK*, *ROS1*, *MET*, *BRAF*, *RET,* and *NTRK*. Kinase fusions have been detected at a high frequency in Spitz tumors and are frequently linked to Spitzoid melanomas [145]. TCGA analysis detected fusions in *BRAF* and *RAF1* kinases in melanomas, but did not report fusions in *ALK*, *ROS1*, *MET*, *RET,* or *NTRK,* which are clinically targetable [28,146]. However, other studies identified a subset of melanoma patients with targetable gene fusions, albeit with a rare frequency. For example, a Chinese study identified *ALK* break points in four patients among 30 acral melanomas (13.3%), and no *ALK* fusions were identified in 28 mucosal melanoma patients [147]. Busam et al. did not report *ALK* fusions in a cohort of 600 melanoma patients, but reported *ALK* expression by *IHC* that was detected in 16 samples [148]. Turner et al. reported chromosomal rearrangement in *RET* (a patient with acral melanoma), *BRAF,* and *ROS1* (a superficial spreading melanoma subtype and an unknown primary) in four patients (*n* = 59) [149]. Another recent study using next-generation sequencing (NGS) identified gene fusions in a cohort of 122 patients with a frequency of 2.5% *BRAF* (three cases), 2.5% *NTRK3* (three cases), 0.8% *ALK* (one case), and 0.8% *PRKCA* (one case) [32]. Taken together, these results suggest that gene fusions in melanomas might be subtype-specific and enriched (i.e., most detected cases have been found in acral melanomas) although some fusions like *NTRK* were identified at a very low rate in cutaneous melanomas [150,151]. In addition, the majority of gene fusions were detected in cases where no driver mutations were present, suggesting the oncogenic potential of these chimeric fusions.

The targeted treatment of oncogenic gene fusions is already a standard of care in tumors such as lung cancer. Evidence on the efficacy of these targeted treatments in melanomas harboring structural chromosomal abnormalities is lacking due to the rarity of these alterations, but some evidence suggests a possible role in melanoma. The *NTRK* inhibitor larotrectinib demonstrated clinical efficacy in refractory solid tumors with *NTRK* fusions in a phase 1/2 trial. In this study, a total of 55 patients (including four melanoma patients) were enrolled, and the ORR was 75% with a median duration of responses and a PFS that has not been reached at a median follow up of 9.4 months [152]. A pooled analysis of three phase 1/2 trials of larotrectinib included seven melanoma patients, and they reported three confirmed responses and an ORR of 43%, but information on the duration of the response was not reported on in melanoma patients [153]. Another *NTRK/ROS1/ALK* inhibitor (entrectinib) demonstrated clinical safety and efficacy in phase 1/2 trials, but no melanoma patients were included in these studies [154]. Of interest, one case report demonstrated the efficacy of entrectinib in a patient with an acral melanoma harboring *ROS1* gene fusion [155]. The patient received 600 mg of entrectinib orally daily and achieved a PR within 2 weeks of treatment initiation, and the response was ongoing at 11 months, with side effects that included dyspnea and weight gain. In contrast, entrectinib did not yield a response in another patient with a mucosal vulvar melanoma who had an *ALK* isoform with an alternative transcription initiation (*ALK^ATI^*) who experienced rapid progression and dyspnea with pain after receiving the targeted treatment [156]. Of note, *ALK^ATI^* is not associated with genetic aberrations on the *ALK* locus.

Despite the lack of strong evidence for targeting gene fusions in melanomas, the FDA approval of larotrectinib in solid tumors (including melanomas) with *NTRK* fusions provides a base for further research on the efficacy of other fusion protein inhibitors in a small subset of melanoma patients.

## 7. Targeting Epigenetic Regulators

Epigenetics involves the role of gene expression alterations associated with diseases that are independent of structural DNA changes (i.e., enzymes and proteins that regulate histone function and gene expression through methylation and acetylation) [157]. There is compelling evidence of the oncogenic role of several somatic mutations in epigenetic modifiers in multiple human cancers, including melanomas. Most of these mutations are in genes that code for chromatin modifier proteins [158].

One of the most frequently mutated chromatin regulators occurs in the enhancer of the zeste homolog 2 (*EZH2*) gene, a trimethylator of lysine-27 in histone (H3K27me3), which controls the expression of genes essential to cell cycle regulation, senescence, and apoptosis [159]. Of interest, *EZH2* can function as either an oncogene or a tumor suppressor gene in different cancers for unknown reasons. In melanomas, the most frequent hotspot mutations in *EZH2* are in the enzymatic SET-domain Y641, and the frequency of different *EZH2* mutations in melanomas is approximately 5% [160]. Focal amplifications of *EZH2* were also identified in 5.7% of melanoma cases from TCGA database. Of interest, *EZH2* is suggested to mediate resistance in *BRAF* mutant melanomas, and to cooperate with *BRAF* to maintain tumor progression [161]. In addition, *EZH2* can increase melanoma growth and is associated with a high proliferation rate through the silencing of tumor suppressors [162,163]. The use of EZH2 inhibitors in melanoma cell lines demonstrated anti-tumor efficacy in both *EZH2* wild-types and mutant forms [164]. Tazemetostat is a selective, reversible, small molecule inhibitor of the histone methyl transferase of *EZH2* that can inhibit both wild-type and mutant forms. The medication is FDA approved for the treatment of relapsed follicular lymphomas and advanced epitheloid sarcomas. Currently, an ongoing trial is underway to evaluate the safety and efficacy of adding tazemetostat to dabrafenib and trametenib in progressive metastatic melanoma patients with *EZH2* alterations (NCT04557956).

Isocitrate hydrogynase 1/2 (*IDH1/2*) are a set of enzymes that function within the mitochondria and cytoplasm, respectively, in the TCA cycle to convert isocitrate into a-ketoglutarate to generate *NADPH* from *NADP+,* which is important in cellular metabolism. Somatic point mutations in arginine residues can lead to the altered function of the enzyme, leading to the formation of the oncometabolite D-2-hydroxyglutarate (*D2HG*) which, in turn, can lead to the CpG island promotor and hypermethylation, as well as suppressing the transcription of key gene regulators [165,166]. *IDH* mutations have been identified in several malignancies (especially *IDH1* ^R132^), and were found to be targetable in cancers such as AML and gliomas [165]. Two *IDH* inhibitors, ivosedinib (an *IDH1* inhibitor) and enasedinib (an *IDH2* inhibitor) demonstrated efficacy against gliomas, cholangiocarcinomas, and AML in clinical trials [167,168,169,170]. In melanomas, *IDH* mutations were first described in 2010, and a follow-up analysis of TCGA data identified a frequency of 4.9% [28,171]. However, the presence of *IDH* mutations in melanomas does not appear to have a prognostic impact on survival compared to *IDH* wild-types [165]. Despite the evidence that *IDH* mutations can confer the in vivo growth ability in melanoma cell lines with *BRAF* mutations, the biological implications of *IDH* mutations in melanomas and their impacts on tumorigenesis and progression remain to be elucidated [172]. Currently, an ongoing phase 1 trial is underway to evaluate the safety and efficacy of a novel *IDH1* inhibitor (LY3410738) in advanced solid tumors with *IDH1* mutations, including melanomas (NCT04521686).

The biological impact of alterations in other epigenetic regulators, such as *ARID2* which occurs at a high frequency in melanomas, are lacking, and further research is essential to illustrate their role in melanomagenesis. The main challenge will be integrating epigenetic regulators in the melanoma treatment paradigm, which is dependent on better understanding of the role of epigenetic alterations in tumorigenesis.

## 8. Targeting Homologous Recombination Deficiency and the DNA Damage Response Pathway

**Figure 5:**
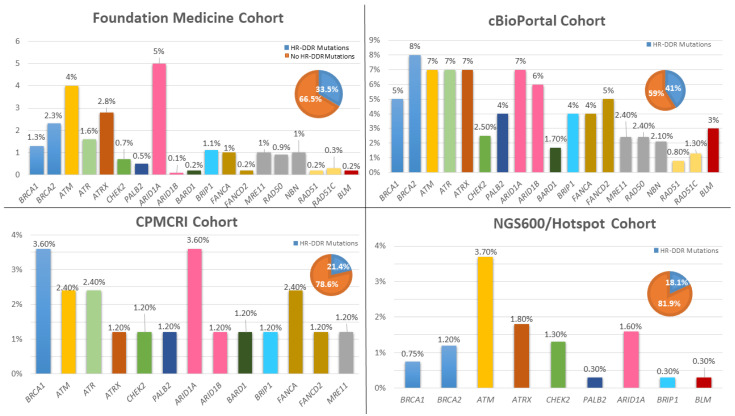
Frequency of commonly mutated genes of the HR-DDR pathway in melanomas from different cohorts including: FoundationOne medicine (*n* = 1986), cBioPortal (1088), CPMCRI (84), and NGS600/Hotspot (*n* = 670) cohorts. Bar charts represent the percentage of specific gene mutations in each cohort. Pie charts represent the percentage of melanoma samples with at least one mutation in the HR-DDR pathway. HR-DDR: Homologous recombination-DNA damage response. Pie charts: blue color represents the percentage of melanoma specimens harboring at least 1 mutant gene from homologous recombination pathway. The orange color represents percentage of melanoma specimens without homologous recombination gene mutations. CPMCRI: California Pacific Medical Center Research Institute. NGS: Next-generation Sequencing.

Genomic integrity is contingent on the presence of effective cellular machinery that can recognize DNA damage caused by reactive oxygen species and spontaneous mutagenesis. Subsequentially, this system operates by recruiting and providing cellular instruments to repair damaged DNA. This is achieved by DNA damage response (DDR) genes that function through two main mechanisms: (1) homologous recombination (HR), and (2) non-homologous end-joining (NHEJ). The advantage of the HR pathway is its high fidelity in DNA repair, as it uses the non-damaged DNA template to repair damaged DNA, but it can only function during the S and G2 phases. In contrast, NHEJ provides an immediate repair mechanism, but is more error prone. The role of genomic instability due to alterations in DDR genes has been well established as an essential process in tumorigenesis and cancer progression in multiple malignancies [173]. More than 400 genes have been identified to play a role in the DDR pathway which can repair double-stranded and single-stranded DNA breaks [173]. Major DDR pathways are functionally grouped into multiple categories that are responsible for base/nucleotide excision repair, direct damage reversal/repair, mismatch repair, and the Fanconi anemia pathway, among others [174]. Of interest, while alterations in DDR genes provide an advantage to cancer cells, they generate vulnerability which, when exploited, can lead to anti-tumor effects. This concept was emphasized through the development of poly (ADP-ribose) polymerase (PARP) inhibitors, which showed safety and efficacy in ovarian, breast, prostate, and pancreatic cancers that harbor defects in HR genes (mainly *BRCA1/2* mutations), which led to their FDA approvals [175,176,177,178,179,180]. Currently approved inhibitors are olaparib, rucaparib, talazoparib, and niraparib, which are orally administered, and exert their effect through the trapping of PARP enzymes, which are essential for single-strand break repair. The efficacy is highly evident in cancer cells with HR disruptions due to *BRCA 1/2* mutations leading to synthetic lethality. The term homologous recombination deficiency (HRD) represents the breach in HR repair mechanisms due to mutations in DDR genes such as *BRCA1/2* and *ATM,* among many others [181]. Several assays have been developed in an attempt to identify the HRD status in cells through the analysis of promiscuous mutations in DDR genes, a LOH, large-scale state transitions, the number of telomeric allelic imbalances, and epigenetic alterations that lead to the silencing of DDR genes [182]. These methods are still under investigation as a biomarker surrogate for the response to PARP inhibitors, as the majority of the approvals of PARP inhibitors are in specific cancers harboring mutations in *BRCA1/2* [183]. The presence of DDR gene mutations has been described in melanomas from TCGA [182]. Heeke et al. identified an HR mutation frequency of 18.1% in 670 melanoma samples [184]. Moreover, studies investigating samples from FoundationOne medicine, cBioPortal, and CPMCRI reported different DDR gene mutation frequencies in melanomas (Figure 5) [185]. The frequency in DDR mutations varies in melanomas in comparison with other cancers. For example, the rate of *BRCA1/2* mutations is lower in melanomas compared to breast, ovarian, and prostate cancers [184]. Conversely, higher rates of ATM mutations are observed in melanomas compared to breast and ovarian cancers [184]. Preclinical evidence exists regarding the possible anti-tumor efficacy of targeting DDR genes in melanoma cells, such as the inhibition of *RAD51* [186]. Recently, the anti-tumor activity of niraparib was demonstrated in melanoma cell lines with mutations in *BRCA1*, *ARID1,B* and *CHD2* [187]. Of interest, there is mounting evidence that *PARP* inhibition can reprogram the tumor microenvironment, leading to enhanced tumor– cell intrinsic immunity. This suggests a possible synergistic role of *PARP* inhibitors with immunotherapy, such as ICI, to enhance anti-tumor immunity [188,189].

To date, there is no evidence available from prospective trials on the role of *PARP* inhibition in melanomas or the combination of *PARP* inhibition with immunotherapy. However, several case reports provide a proof-of-concept for the clinical efficacy of *PARP* inhibitors. For example, Lau et al. reported a PR in a melanoma patient with a *PALB2* mutation who progressed during treatment with ICI and was later treated with a PARP inhibitor [190]. In contrast, two case reports suggested possible synergism between olaparib and nivolumab in two melanoma patients who were immunotherapy-refractory patients and who had evidence of HRD [191,192]. Of importance, one patient developed hepatitis as a side effect during combination treatment, which raises a safety concern. Currently, several prospective trials are ongoing to assess the safety and efficacy of combination treatment (*PARP* inhibitors with ICI or *PARP* inhibitor monotherapy) in patients with HRD melanomas (NCT03925350; NCT04187833; NCT04633902). Finally, novel inhibitors of other DDR gene alterations are currently undergoing early phase trials to assess their safety and efficacy in melanomas with HRD (i.e., inhibitors of *ATR*, *ATM, CHK1/2,* and *WEE1,* among others) [183].

## 9. Conclusions

The considerable advances in understanding molecular alterations in melanomas have opened the door for extensive research on novel individualized therapeutic approaches. Distinguishing driver and passenger mutations in melanomas and their implications in melanomagenesis is important for the development of effective targeted therapies. Immune checkpoint inhibitors remain the mainstay of treatment for unresectable, locally advanced, and metastatic melanomas, irrespective of a presence of a biomarker. Targeted therapy including *BRAF* and *MEK* inhibitors and immune checkpoint inhibitors are the standard of care for unresectable locally advanced and metastatic melanomas with *BRAF^V600E/K^* mutations. The innovative high-resolution technology and integration of next-generation sequencing in cancer characterization have allowed for the identification of melanoma driver mutations and have led to the integration of alternative targeted therapies such as tyrosine kinase inhibitors in *c-KIT* relapsed and refractory melanomas. In contrast, common mutations in melanomas such as *NRAS* and *NF1* remain unable to be targeted to date. Current research efforts are focusing on understanding the interplay between specific somatic mutations, their downstream signaling pathways, and other signaling networks. Part of the investigational effort focuses on targeting downstream effectors or parallel pathways that are essential for *NRAS* and *NF1* mutant melanomas, and another investigation is focused on the combined targeted therapy approach. The surge of multiple effective targeted therapies in non-melanoma cancers warrants the rational and cautious investigation of their safety and efficacy in a subset of melanoma patients harboring the specific desired molecular alterations, as they could prove useful. Moreover, there is a need for further investigation into the role of epigenetic changes and DNA damage response alterations/homologous recombination deficiencies in tumorigenesis, aggressiveness, and the resistance to treatment in melanomas. Finally, it is important to note that inclusion of melanoma patients in well-designed clinical trials testing targeted therapies should be emphasized whenever possible to provide a level of evidence on their safety and efficacy.

## Figures and Tables

**Table 1:** Clinical trials and retrospective studies of targeted therapy in *c-KIT* mutant melanoma.

Study Design	Study Drug	Dose	Number of Patients	Type of Mutations	Special Characteristics	Overall Response Rate	Duration of Response	Median Progression Free Survival	Median Overall Survival	Reference
Single group, open label, multicenter, phase 2 trial	Imatinib	400 mg twice daily	28	Mutation and/or amplification	328 patients screened, 51 had *KIT* alterations, 28 were treated, 25 analyzed (3 excluded due to toxicity)	16%	2 CR (94 and 95 weeks), 2 PR (53 and 89 weeks) and 2 transient PR (12 and 18 weeks)	12 weeks	46.3 weeks	[60]
Single arm, open label, single center, phase 2 trial	Imatinib	400 mg daily	43	Mutation and/or amplification	Dose allowed to be escalated to 600 mg or 800 mg daily if POD	22%	Not available	3.5 months	14 months	[61]
Single arm, open label, multicenter, phase 2 trial	Imatinib	400 mg daily	25	Mutation and/or amplification	Dose escalated to 400 mg twice daily if no response	29% (21% excluding non-confirmed responses)	Not available	3.7 months	12.5 months	[62]
Retrospective chart review, single center	Imatinib	400 mg daily	78	Mutation and/or amplification	Largest study, but retrospective	21.8%	Not available	4.2 months	13.1 months	[63]
Single arm, open label, single center, phase 2 trial	Nilotinib	400 mg twice daily	11	Mutation and/or amplification	Of the 11 patients, 9 were evaluated for a response. Most patients had M1c disease	22.2%	2 w/*KIT* mutations responded for 8.4 and 10.0 months. 1 w/amplification had SD for 6 months	2.5 months	7.7 months	[64]
Phase 2 trial with 2 study groups, open label	Nilotinib	400 mg twice daily	19	Mutation and/or amplification	20 enrolled, 19 treated. 4 were not evaluated for radiographic responses to therapy. 2 cohorts: (A) those refractory or intolerant to a prior *KIT* inhibitor; and B) those with brain metastases	10.5% overall (18.1% in cohort A, 2 PR; 0 in cohort B)	One patient in cohort A had ongoing response for 34.5 months. One in cohort B had a 3.9-month response in CNS disease	3.3 months	9.1 months	[65]
Open label, single arm, multicenter, phase 2	Nilotinib	400 mg twice daily	42	Mutation and/or amplification	176 patients screened, 42 enrolled. PFS 8.5 months in CR/PR/SD vs. 7 weeks in PD	16.7%	34 weeks	3.3 months	11.9 months	[66]
Open label, single arm, multicenter, phase 2	Nilotinib	400 mg twice daily	42	Mutation only	First to evaluate nilotinib without prior *KIT* inhibitor therapy. Originally designed for 2 groups (prior dacarbazine vs. nilotinib), but not enough patients	26.2%	7.1 months	4.2 months	18.0 months	[67]
Open label, single arm, multicenter, phase 2	Nilotinib	400 mg twice daily	25	Mutation and/or amplification	4 patients exhibited durable response, 3 persisting (3.6 and 2.8 years for 2 patients with stage IIIC and 2.5 years for 1 with IVM1b)	20%	In patients with CR and PR, 46.8 months	6.0 months	13.2 months	[68]
Two-stage, open label, single arm, single center, phase 2	Dasatinib	70 mg twice daily	30 in stage 2	Mutation only	2 stages: (1) both *KIT*+ and *KIT*-wt w/ mucosal, acral and CSD melanoma; 57 patients, 51 analyzed; (2) trial amended for *KIT*+ only and added vulvo-vaginal and excluded CSD melanoma; 30 patients, 22 analyzed	18.2% in stage 2 5.9% in stage 1	4.2 months in stage 2	2.1 months (stage 1 and 2 combined) 2.7 months in KIT+ patients (both stages)	7.5 months (stage 1 and 2 combined) 11.8 months in *KIT*+ patients (both stages)	[69]
Open label, single arm, single center, phase 2	Sunitinib	50 mg daily	12 (10 analyzed)	Mutation, amplification or over-expression	90 enrolled, 12 treated with sunitib (*KIT* alterations). Sunitib given 4 weeks on, 2 off. Dose reduced to 37.5 or 25 mg if AEs	40%	1 CR for 15 months, 2 PR (1 month and 7 months) in patients with mutations. 1 PR in amplification or over-expression.	Not available	Not available	[70]

Abbreviation: POD: progression of disease; KIT: c-KIT receptor; CR: complete response; PR: partial response; CNS: central nervous system; CSD: chronic sun damage; PFS: progression-free survival; PD: progressive disease; SD: stable disease; w/: with; c-KIT: c-KIT receptor; KIT+: positive for c-KIT receptor.

**Table 2:** Summary of ongoing targeted therapy trials in *NRAS* mutant melanomas.

Drug(s)	Study Phase	Target Population	Sample Size	ClinicalTrials.gov (Accessed on 1 September 2021) Registration
*MEK* + Autophagy inhibitor Trametinib + Hydroxychloroquine	Phase 1b/2	Metastatic or locally advanced unresectable *NRAS* melanoma	29	NCT03979651
*MEK* inhibitor Binimetinib	Phase 2	*BRAF* or *NRAS* mutant locally advanced or metastatic melanoma	183	NCT01320085
*MEK* inhibitor HL-085	Phase 1/2	Stage III or Stage IV *NRAS* mutated melanoma	54	NCT03973151
*MEK* inhibitor FCN-159	Phase 1a/1b	*NRAS* aberrant and *NRAS* mutated melanoma	37	NCT03932253
*RAF* inhibitor + *ERK* inhibitor +/− other agents Encorafenib + LY3214996	Phase 1	Part B: *BRAF* mutant metastatic melanoma refractory or relapse, *NRAS* metastatic melanoma, *BRAF* mutant *NSCLC*	245	NCT02857270
*ERK* inhibitor ASN007	Phase 1	*NRAS/BRAF* mutant melanoma	49	NCT03415126
*ARF*-sparing inhibitor of *BRAF* and *CRAF* + *ERK* inhibitor or *MEK* inhibitor or *CD4/6* inhibitor LXH254 + LTT462 or Trametinib or Ribociclib	Phase 1b	*KRAS/BRAF NSCLC*	331	NCT02974725
	Phase 2	*NRAS* mutant melanoma	320	NCT04417621
ERBB3 antibody + *MEK* inhibitor CDX-3370 + Trametinib	Phase 1b/2 Terminated	*NRAS* melanoma	3	NCT03580382
*RAF* Inhibitor + *MEK* inhibitor + immune check point inhibitor	Phase 1b	Stage IV or Stage III *NRAS* melanoma relapse after check point inhibitor	83	NCT04835805

NRAS: neuroblastoma RAS viral oncogene; BRAF: BRAF gene; NSCLC: non-small cell lung cancer; MEK: mitogen-activated protein kinase; RAF: RAF gene; ERK: extracellular signal-regulated kinase; CRAF: CRAF gene.

## Abbreviations

ALK: Anaplastic lymphoma kinase; AML: Acute myeloid leukemia; ARID2: AT-Rich Interaction Domain 2; BRAF: B-rapidly accelerated fibrosarcoma; cAMP: Cyclic adenosine 3′,5′-cyclic monophosphate; CAPN1: Calpain1; CCND1: cyclinD1; CDKN2A: cyclin dependent kinase inhibitor 2A; CNS: Central nervous system; CNV: Copy number variation; CR: Complete response; CRAF: Proto-oncogene c-RAF; CTLA-4: Cytotoxic T-lymphocyte associated antigen-4; DDR: DNA-damage response; D2HG: D-2-hydroxyglutarate; E2F: E2 Transcription Factor; ERBB3: Erb-B2 Receptor Tyrosine Kinase 3; EZH2: Enhancer of zeste homolog 2; FDA: Food and Drug Administration; FT: Farnesyltransferase; GIST: Gastrointestinal stromal tumor; GOF: Gain of Function; HR: Homologous recombination; HRAS: Harvey rat sarcoma viral oncogene homolog; HRD: Homologous recombination deficiency; ICI: Immune checkpoint inhibitor; IDH1/2: Isocitrate dehydrogenase 1/2; IHC: Immunohistochemistry; JAK: Janus kinase; KIT: Mast/Stem cell growth factor receptor kit; KRAS: Kirsten rat sarcoma viral oncogene homolog; LOF: Loss of function; LOH: Loss of heterozygosity; MAPK: Mitogen-activated protein kinase; MAP2K1: mitogen-activated protein kinase kinase 1; MAP2K2: mitogen-activated protein kinase kinase 2; MDM2: Mouse double minute-2; MDMX: Murine double minute X; MDS: Myelodysplastic syndrome; MEK: Mitogen-activated protein kinase kinase; MET: Mesenchymal–epithelial transition factor; mTOR: mammalian target of rapamycin; NF1: Neurofibromatosis type 1; NGS: Next-generation sequencing; NHEJ: Non-homologous end-joining; NRAS: Neuroblastoma RAS viral oncogene homolog; NTRK: Neurotrophic tyrosine receptor kinase; ORR: Objective response rate; OS: Overall survival; PARP: Poly (ADP-ribose) polymerase; PD1: Programmed death-1; PDGFR: Platelet-derived growth factor receptor; PDL1: Programmed death ligand-1; PFS: Progression-free survival; PI3K: Phosphatidylinositol 3-kinase; PKA: Promotes protein kinase A; PLC: Phospholipase C; PR: Partial response; PRKCA: Protein kinase C α; PTEN: Phosphatase and TENsin homolog; PTPN11: Tyrosine-protein phosphatase non-receptor type 11; RAC1: Ras-related C3 botulinum toxin substrate 1; RB: Retinoblastoma; RTK: Receptor tyrosine kinase; SCF: Stem cell factor ligand; SD: Stable disease; SEER: Surveillance, Epidemiology, and End Result Program.; SOS1: Son of sevenless 1; STAT: Signal transducers and activators of transcription; STK19: Serine/threonine-protein kinase 19; TCA: Tricarboxylic acid; TCGA: The Cancer Genome Atlas; TERT: Telomerase Reverse Transcriptase; TKI: Tyrosine kinase inhibitor; TP53: Tumor protein 53.
